# Integrative Analysis of Whole-Genome and Transcriptomic Data Reveals Novel Variants in Differentially Expressed Long Noncoding RNAs Associated with Asthenozoospermia

**DOI:** 10.3390/ncrna11010004

**Published:** 2025-01-14

**Authors:** Maria-Anna Kyrgiafini, Maria Katsigianni, Themistoklis Giannoulis, Theologia Sarafidou, Alexia Chatziparasidou, Zissis Mamuris

**Affiliations:** 1 Laboratory of Genetics, Comparative and Evolutionary Biology, Department of Biochemistry and Biotechnology, University of Thessaly, Viopolis, Mezourlo, 41500 Larissa, Greece; 2 Laboratory of Biology, Genetics and Bioinformatics, Department of Animal Sciences, University of Thessaly, Gaiopolis, 41336 Larissa, Greece; 3 Embryolab IVF Unit, St. 173-175 Ethnikis Antistaseos, Kalamaria, 55134 Thessaloniki, Greece

**Keywords:** asthenozoospermia, male infertility, ncRNA, lncRNA, biomarker, variant

## Abstract

**Background/Objectives**: Asthenozoospermia, characterized by reduced sperm motility, is a common cause of male infertility. Emerging evidence suggests that noncoding RNAs, particularly long noncoding RNAs (lncRNAs), play a critical role in the regulation of spermatogenesis and sperm function. Coding regions have a well-characterized role and established predictive value in asthenozoospermia. However, this study was designed to complement previous findings and provide a more holistic understanding of asthenozoospermia, this time focusing on noncoding regions. This study aimed to identify and prioritize variants in differentially expressed (DE) lncRNAs found exclusively in asthenozoospermic men, focusing on their impact on lncRNA structure and lncRNA–miRNA–mRNA interactions. **Methods**: Whole-genome sequencing (WGS) was performed on samples from asthenozoospermic and normozoospermic men. Additionally, an RNA-seq dataset from normozoospermic and asthenozoospermic individuals was analyzed to identify DE lncRNAs. Bioinformatics analyses were conducted to map unique variants on DE lncRNAs, followed by prioritization based on predicted functional impact. The structural impact of the variants and their effects on lncRNA–miRNA interactions were assessed using computational tools. Gene ontology (GO) and KEGG pathway analyses were employed to investigate the affected biological processes and pathways. **Results**: We identified 4173 unique variants mapped to 258 DE lncRNAs. After prioritization, 5 unique variants in 5 lncRNAs were found to affect lncRNA structure, while 20 variants in 17 lncRNAs were predicted to disrupt miRNA–lncRNA interactions. Enriched pathways included Wnt signaling, phosphatase binding, and cell proliferation, all previously implicated in reproductive health. **Conclusions**: This study identifies specific variants in DE lncRNAs that may play a role in asthenozoospermia. Given the limited research utilizing WGS to explore the role of noncoding RNAs in male infertility, our findings provide valuable insights and a foundation for future studies.

## 1. Introduction

Infertility is an increasingly prevalent issue worldwide, affecting approximately 15% of couples attempting to conceive [1], which amounts to an estimated 72.4 million individuals globally [2]. The male factor contributes to 50% of all infertility cases [3], and male infertility is defined by the World Health Organization (WHO) as a male’s inability to induce pregnancy in a fertile female after one year of regular, unprotected sexual intercourse. Several factors contribute to male infertility, including hormonal imbalances, anatomical abnormalities, genetic disorders, and environmental influences [4]. Among these factors, sperm quality is particularly crucial, with sperm count, motility, and morphology serving as key indicators of male reproductive health [5]. One notable condition related to male infertility is asthenozoospermia, characterized by reduced sperm motility, which significantly affects fertility outcomes. More specifically, according to the fifth edition of the WHO guidelines (https://apps.who.int/iris/handle/10665/44261, accessed on 17 November 2024), asthenozoospermia is defined as having sperm motility below 40% or progressive motility below 32%. Thus, while spermatozoa may be present in the semen, they lack the necessary movement to travel from the vagina to the fallopian tube, making fertilization unattainable. Asthenozoospermia is a major contributor to male infertility, with a prevalence of approximately 18.71% among infertile men [6]. This condition is related to a variety of genetic, metabolic, and environmental factors, emphasizing the complexity of its underlying mechanisms [7].

Noncoding RNAs (ncRNAs) have emerged as critical regulators of gene expression, playing an essential role in various cellular processes, including those required for male fertility [8]. In the context of male infertility, ncRNAs such as microRNAs (miRNAs), long noncoding RNAs (lncRNAs), and circular RNAs (circRNAs) are gaining prominence for their involvement in spermatogenesis, sperm motility, and overall sperm quality. MiRNAs, the most studied type of ncRNAs, are small single-stranded RNA molecules that regulate gene expression post-transcriptionally by binding to the 3′ untranslated regions (UTRs) of target mRNAs, leading to their degradation or translational repression [9]. CircRNAs, a newly characterized class of ncRNAs with a covalently closed-loop structure, have gained attention for their roles in male infertility [10]. CircRNAs are expressed in the human testes, spermatozoa, and seminal plasma [11]. These molecules act as miRNA sponges, regulators of RNA-binding proteins, and modulators of transcription, contributing to the regulation of key molecular pathways [12]. Recent studies have highlighted their involvement in the asthenozoospermic phenotype, where they are believed to affect pathways related to mitochondrial function and sperm motility [11]. Furthermore, circRNAs have been implicated for their characteristics, such as their high stability, that allow them to be used as biomarkers [13]. Thus, the intricate interplay among these ncRNA classes forms complex regulatory networks that are essential for maintaining sperm health, and disruptions in these networks may underlie various forms of male infertility.

Recent advances in molecular techniques and next-generation sequencing (NGS) have increasingly highlighted the critical role of long noncoding RNAs (lncRNAs) in asthenozoospermia. Research has shown that lncRNAs participate in various regulatory networks that affect sperm motility and spermatogenesis, highlighting their potential as biomarkers and therapeutic targets for infertility [14]. Several studies have also investigated the differential expression of lncRNAs in the semen of asthenozoospermic versus normozoospermic men, providing insight into the underlying regulatory mechanisms [15,16]. Beyond the importance of studying these expression profiles, genetic variants also substantially impact the structure, function, and gene regulation of lncRNAs [17]. These variations, which include alternative splicing and single-nucleotide polymorphisms (SNPs), can lead to the production of diverse lncRNA isoforms, each with distinct biological functions. Through alternative splicing, lncRNAs can generate multiple isoforms, each possessing unique regulatory roles in cellular processes [18]. The differential expression of specific lncRNA splice variants has been linked to various diseases, including cancer, emphasizing their critical role in disease mechanisms (reviewed in Khan et al. (2023) [18]). SNPs, meanwhile, can alter the secondary structure of lncRNAs, impacting their interactions with RNA-binding proteins (RBPs) and other molecules, such as microRNAs (miRNAs), and subsequently influencing gene expression pathways [19,20]. As a result, structural variations caused by SNPs may disrupt the binding and recruitment functions of lncRNAs, potentially contributing to complex diseases [20]. Therefore, genetic variations in lncRNAs can affect their regulatory functions, impacting key biological processes [21]. Although these variants broaden the functional repertoire of lncRNAs, they can also lead to dysregulation, contributing to disease pathology. Understanding these dynamics is crucial for elucidating the mechanisms behind male infertility and for developing therapeutic strategies that target lncRNAs.

However, research indicates a significant knowledge gap regarding the role of variants in lncRNAs and their association with male infertility. While recent studies have identified various lncRNAs linked to male infertility, the specific impact of genetic variants within these lncRNAs remains underexplored. Specifically, a systematic review highlights that many lncRNAs are deregulated in male infertility, yet their mechanisms and the influence of genetic variants are not well understood [15]. Furthermore, building on previous studies that explored the role of mRNAs and their differential expression in asthenozoospermia [22], as well as the predictive value of oxidative phosphorylation coding variants in asthenozoospermia [23], we undertook this study to complement those findings. Coding regions have a well-characterized role and a predictive value for asthenozoospermia, but our aim is to provide a more holistic understanding by focusing on the noncoding regions of the genome, offering additional insights into its underlying mechanisms.

Therefore, the objective of this study was to identify variants in lncRNAs that are likely to contribute to male infertility by examining unique genetic variants that may alter critical functions of the lncRNAs. Using whole-genome sequencing (WGS) data, we identified variants that were present exclusively in asthenozoospermic men. From these, we selected variants that mapped specifically to differentially expressed (DE) long noncoding RNAs (lncRNAs). We then prioritized these variants based on their functional significance, their potential impact on the lncRNA structure, and disruption of lncRNA–miRNA interactions using in silico analysis. Finally, we conducted gene ontology and pathway analyses to identify the molecular mechanisms and pathways affected by these disrupted interactions. This approach provides new insights into the role of lncRNAs in asthenozoospermia and aims to serve as a valuable reference for future research, as the identified variants may hold significant importance in unraveling the genetic basis of asthenozoospermia.

It should be noted, however, that the primary goal of this study is to explore the potential of lncRNAs as biomarkers or regulators in asthenozoospermia rather than to establish causal relationships, since male infertility is a complex condition with both coding and noncoding genes contributing to the phenotype [24]. By prioritizing ncRNA candidates, this study aims to highlight their potential roles in the condition and provide a basis for further functional investigations. The results of this study complement existing knowledge on coding sequences while expanding our understanding of the role of noncoding RNA in male infertility.

## 2. Results

### 2.1. Whole-Genome Sequencing—RNA Sequencing and Combination of Datasets

Following whole-genome sequencing, an in-depth data analysis compared genetic variants between normozoospermic and asthenozoospermic individuals to identify exclusive variants in each group. The results showed that 680,099 variants were unique to asthenozoospermic individuals, mapping to 26,019 genes, while normozoospermic individuals exhibited 2,329,803 unique variants in 30,362 genes. For this study, only the variants exclusive to asthenozoospermic individuals were selected for further investigation, as the aim was to detect and investigate variants in lncRNA regions that contribute to male infertility and have the potential to be used as biomarkers.

Regarding RNA seq analysis, Lu et al. (2020) [22] examined the expression profiles of lncRNAs in seminal plasma exosomes, identifying 995 differentially expressed lncRNAs (DE lncRNAs) between the two groups (*p*-value ≤ 0.05, absolute log2 fold change (FC) ≥ 1). More specifically, they identified 656 upregulated and 339 downregulated lncRNAs in asthenozoospermia compared to the normozoospermic control group (Table S1).

**Figure 1:**
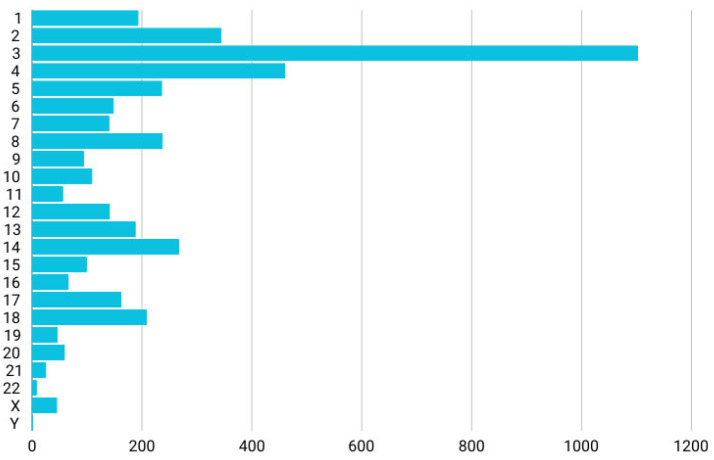
Chromosomal distribution of unique variants found on DE lncRNAs in asthenozoospermic men. The *x*-axis represents the chromosomes, while the *y*-axis shows the number of variants.

Subsequently, by integrating these two datasets, we focused on unique variants found only in asthenozoospermic men, specifically those located in DE lncRNAs according to the study by Lu et al. (2020) [22]. These variants were chosen for their potential to alter lncRNA structure and function, which could influence gene regulation and contribute to the male infertility phenotype. Using this approach, we identified 4173 unique variants mapped to 258 lncRNAs. The complete list of these unique variants mapped onto DE lncRNAs can be found in Table S2. The chromosomal distribution showed that the highest number of these lncRNA variants was located on chromosome 3 (26%), followed by chromosome 4 (11%). Fewer variants were observed on the sex chromosomes, with 46 variants (1.1%) on the X chromosome and only 2 variants on the Y chromosome, as illustrated in Figure 1. These variants were then prioritized by applying a series of filters to investigate their role in asthenozoospermia.

### 2.2. Prioritized Variants with Functional Significance

After identifying unique variants specific to asthenozoospermic men that mapped to differentially expressed long noncoding RNAs (DE lncRNAs), we aimed to prioritize these variants based on their functional relevance. This step involved a thorough investigation using two key databases: RegulomeDB 2.2 [25] and the 3DSNP v2.0 database [26].

RegulomeDB 2.2 is a comprehensive database that annotates variants based on their potential regulatory roles. The database ranks variants from 1a (highest potential for a regulatory role) to 7, indicating the strength of the evidence for a variant’s regulatory impact. In this study, we focused on variants with RegulomeDB ranks between 1a and 2c, representing those most likely to be functionally impactful. Additionally, 3DSNPscore provides scores based on the three-dimensional interactions of SNPs within the genome. A 3DSNPscore greater than 20 was used as our criterion to identify variants with a probable functional role and an impact on gene regulation.

Applying these criteria (3DSNP score > 20 and RegulomeDB rank between 1a and 2c) allowed us to identify 144 prioritized variants with potential functional significance. Notably, among these variants, 32 (22.2%) had a 3DSNP score exceeding 100, indicating a strong regulatory role. The prioritized variants, along with their RegulomeDB rank and 3DSNP score, are presented in Table S3.

### 2.3. Prioritized Variants with an Impact on lncRNA Structure

After prioritizing variants based on their functional significance, we further filtered them to identify those that directly impact lncRNA structure. Using the lncRNASNP v3 database [27], we examined structural changes caused by variants. It is known that even minor changes in nucleotide sequences can alter the thermodynamic stability of RNA, which in turn influences the three-dimensional conformation and overall stability of lncRNAs [17]. Such structural modifications can disrupt lncRNA interactions with other molecules, including proteins and DNA, potentially interfering with their regulatory functions [19]. Furthermore, since lncRNAs do not code for proteins, maintaining their structural integrity is crucial for their proper functioning. Variants that compromise this integrity can significantly impair lncRNA functionality and alter the roles of lncRNAs in cellular processes and gene regulation [17,19]. Therefore, we prioritized SNPs with predicted impact on lncRNA structure in our study to identify variants potentially involved in male infertility.

To filter the results, we selected variants with a *p*-value < 0.2, a cutoff indicating structural impact as determined by lncRNASNP v3 [27]. By applying this criterion, we identified five variants that affect the structure of five lncRNAs (Table 1). This subset of prioritized variants represents those with the highest likelihood of influencing lncRNA stability and function, providing insight into their potential role in male infertility and regulatory disruption.

### 2.4. Prioritized Variants Disrupting lncRNA–miRNA Interactions and Affected Molecular Mechanisms and Pathways

In this study, we prioritized unique variants mapped to differentially expressed long noncoding RNAs (DE lncRNAs) in asthenozoospermic men to evaluate their role in male infertility. After identifying variants with a potential functional role, we further refined our focus to those variants that could also disrupt interactions between lncRNAs and microRNAs (miRNAs), as such disruptions could significantly impact gene regulation pathways relevant to sperm function and male fertility.

Using the lncRNASNP v3 database [27], we identified variants that affect miRNA–lncRNA interactions, leading to the gain or loss of binding sites on DE lncRNAs. Through this analysis, we found 20 variants across 17 lncRNAs that impacted interactions with 110 miRNAs. The complete list of variants and their corresponding affected interactions (lncRNAs and miRNAs) can be found in Table S4. Subsequently, these disrupted interactions were further analyzed to understand the molecular pathways and biological mechanisms potentially influenced by these variations. At first, to identify the gene targets of these miRNAs, we used miRTargetLink 2.0 [28], focusing exclusively on experimentally validated strong interactions. This approach allowed us to construct a reliable network of gene targets, offering insights into the molecular pathways affected by the lncRNA–miRNA interactions disrupted by variants in lncRNAs. The complete list of these genes and their interactions with miRNAs is provided in Table S5.

**Figure 2:**
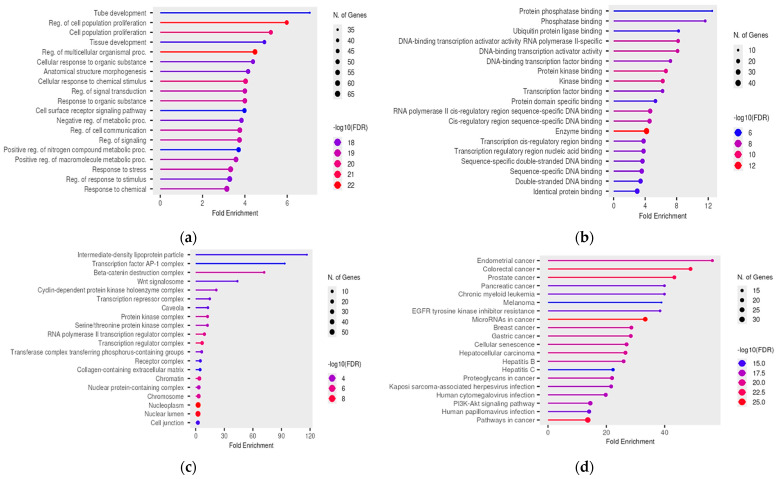
Statistically significant (**a**) GO biological process, (**b**) GO molecular function, (**c**) GO cellular component, (**d**) KEGG pathway terms associated with the gene targets of the miRNAs that are affected by variants in DE lncRNAs (miRNA–lncRNA interaction disruption). The size and color of the dots represent the number of genes and the range of statistical significance, respectively. The *y*-axis represents biological terms, and the *x*-axis, the fold enrichment. The *p*-values were corrected for multiple tests using the false discovery rate (FDR).

Furthermore, to elucidate the biological mechanisms and pathways affected by these disruptions, we conducted gene ontology (GO) [29,30] analyses and Kyoto Encyclopedia of Genes and Genomes (KEGG) [31] pathway analyses on the aforementioned gene targets using ShinyGO 0.81 [32]. These analyses revealed dysregulation in processes such as cell proliferation, tissue development, regulation of metabolism, and signaling pathways. According to KEGG pathway analyses, many miRNA target genes were involved in pathways associated with various types of cancer as well. In terms of cellular components, the most enriched categories included the transcription factor AP-1 complex, the β-catenin destruction complex, and the Wnt signalosome, while the most enriched category for molecular function included genes associated with phosphatase binding (Figure 2a–d).

## 3. Discussion

Long noncoding RNAs (lncRNAs) have emerged as key regulators of gene expression, playing crucial roles in various biological processes, including spermatogenesis and sperm function [33,34]. Unlike protein-coding genes, lncRNAs often function through complex mechanisms, such as modulating chromatin structure, interacting with proteins, regulating transcription, and acting as molecular sponges for microRNAs (miRNAs) [35,36]. Variants within lncRNA sequences can disrupt their structure, stability, and interactions, affecting their functionality and potentially leading to the dysregulation of critical gene networks essential for male reproductive health. However, there is a knowledge gap regarding the impact of variants on lncRNAs and male infertility.

**Figure 3:**
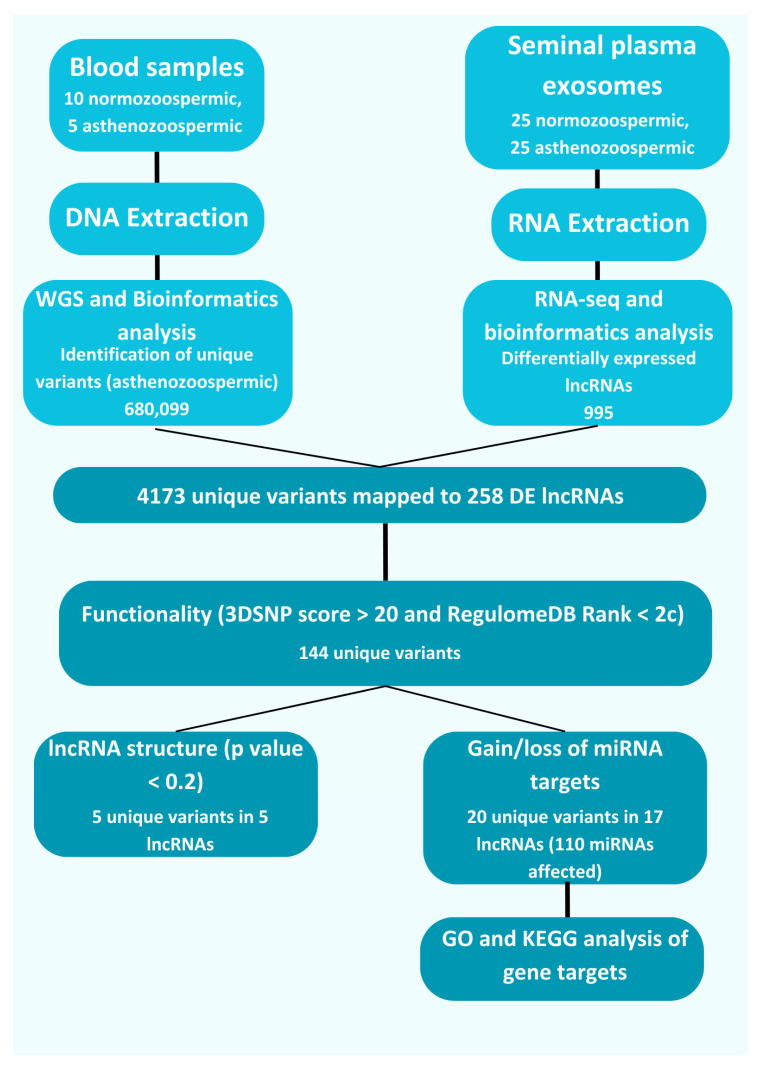
Flow chart of the study methodology and findings.

In this study, we integrated whole-genome sequencing (WGS) and RNA-sequencing (RNA-seq) data to uncover unique genetic variants associated with asthenozoospermia, focusing on those mapped to differentially expressed lncRNAs. To prioritize these variants, we employed a three-step in silico analysis pipeline. First, we identified variants with potential functional regulatory roles. Next, we focused on variants predicted to affect the secondary structure of lncRNAs and their interactions with microRNAs (miRNAs). Finally, we explored the downstream effects by identifying the target genes of the affected miRNAs and conducting gene ontology (GO) and KEGG pathway enrichment analyses. Our findings are summarized in Figure 3.

### 3.1. Variants Affecting lncRNA Structure

Variants within lncRNA sequences, particularly single-nucleotide polymorphisms (SNPs), can disrupt their structural conformation, altering base pairing patterns and potentially affecting the stability and function of the lncRNA [19]. These structural changes may also impact binding sites for RNA-binding proteins (RBPs), which are crucial for modulating transcriptional and post-transcriptional pathways [19,37]. Consequently, such variants could contribute to the dysregulation of gene networks essential for normal sperm development and motility.

In this study, we prioritized variants with functional significance predicted to affect the structural integrity of differentially expressed lncRNAs in asthenozoospermic samples. Notably, none of the identified variants have previously been associated with male infertility or any other diseases, highlighting their potential novelty and significance in this context. Among the affected lncRNAs, *NKX2-1-AS1* has been linked to cancer progression and plays a role in tumor development [38,39,40]. *RUVBL1-AS1* is less studied but has been tentatively associated with leukemia [41]. *NEXN-AS1* is another notable lncRNA, known for its role in mitigating atherosclerosis [42,43]. In contrast, lnc-*AGPAT5-1* and *LINC01914* remain largely understudied, with no relevant literature available, suggesting a potential area for future investigation.

### 3.2. Variants Affecting lncRNA–miRNA Interactions

miRNA–lncRNA interactions are key regulators of gene expression, playing pivotal roles in numerous biological processes. One prominent mechanism involves lncRNAs acting as molecular “sponges”, sequestering miRNAs and reducing their availability to bind to target mRNAs [44,45]. This modulation influences the regulatory effects of miRNAs on gene expression, potentially altering expression patterns associated with various disease states. Moreover, genetic variants within lncRNAs can disrupt these interactions by altering the binding affinity between lncRNAs and miRNAs, leading to the gain or loss of miRNA–lncRNA binding [46]. Such disruptions can have significant downstream effects on gene regulation, contributing to disease development and abnormalities [47], including male infertility.

In this study, we prioritized functionally significant variants located in differentially expressed (DE) lncRNAs that are predicted to disrupt interactions between lncRNAs and miRNAs in asthenozoospermia. Notably, the variant rs2969359 has previously been identified as unique to teratozoospermia and is mapped to a lncRNA that is differentially expressed between normozoospermic and teratozoospermic individuals [48]. Although no other variants in this analysis have been previously associated with male infertility, rs2969359 shows promise as a potential biomarker. Therefore, further research is needed to validate its utility and investigate its role.

Regarding the lncRNAs on which the variants are mapped, as previously, most have been associated with cancer. For example, *THUMPD3-AS1* is an autophagy-related lncRNA linked to several cancers [49,50], including prostate and bladder cancer [51,52,53]. Similarly, *NKX2-1-AS1* [38,39,40] and *PSMA3-AS1* [54,55] have strong associations with cancer, with *PSMA3-AS1* also implicated in preterm delivery [56,57]. *ZBED5-AS1* [58] and *LINC02600*, the latter being an oxidative-stress-related lncRNA [59,60], are also primarily linked to cancer. *LINC01405* [61,62] and *LINC00667*, an oncogenic lncRNA [63], have also been consistently reported in cancer studies. Furthermore, *ZNF503-AS1* is related to genome instability and cancer [64,65], while *LINC01116* has a well-established role in cancer biology, promoting cell proliferation, invasion, and migration and inhibiting apoptosis [66]. Interestingly, however, *LINC01116* has also been associated with polycystic ovary syndrome (PCOS) [67] and teratozoospermia [48]. The lncRNA *AATBC* (Apoptosis Associated Transcript in Bladder Cancer) has been studied for its role in cancer [68,69] but is also involved in mitochondrial function [70]. *AIRN*, another lncRNA, plays a significant role in gene regulation and development across various species, particularly in mammals. It is known for its involvement in genomic imprinting, as it is imprinted and expressed paternally [71]; chromatin architecture modification [72]; and cancer [73]. Finally, *UCHL1-DT*, *LINC02018*, and *lnc-AGPAT5-1* are less characterized, with no significant findings reported regarding their functional roles to date.

Subsequently, we identified the gene targets of miRNAs affected by variants in lncRNAs, resulting from the disruption of lncRNA–miRNA interactions, and examined their functional roles. Gene ontology (GO) molecular function analysis revealed that many of these gene targets are involved in phosphatase binding, a process with significant implications for male infertility. Spermatogenesis is a highly complex process that involves the differentiation of spermatogonia into mature spermatozoa and is tightly regulated by the balance between protein phosphorylation and dephosphorylation [74]. The phosphorylation of sperm proteins is linked to male fertility, as this post-translational modification is crucial in all stages of sperm cell development, contributing to sperm differentiation, maturation, and function [75]. Various phosphatases, such as protein phosphatase 1 (PP1) and PTPN11, are critical regulators of cellular processes necessary for spermatogenesis and sperm motility [76,77]. Notably, mutations in the *PSPH* gene have been associated with severe male infertility, particularly oligoasthenozoospermia (OA), a condition characterized by low sperm count and reduced motility [78]. Furthermore, the sperm-specific isoform PP1γ2 is known to bind to several key proteins within sperm cells, playing a pivotal role in processes such as motility and capacitation. Dysregulated expression or activity of PP1γ2 has been linked to asthenozoospermia [76]. Overall, our findings align with previous studies that underscore the importance of phosphorylation and its regulation in male fertility, supporting the notion that defects in this process can adversely affect sperm motility [74].

KEGG pathway analysis also revealed a significant association between gene targets of affected miRNAs and cancer, further strengthening the established link between male infertility and cancer. Previous studies have indicated that infertile men are at a higher risk of developing cancer, suggesting the presence of shared molecular pathways [79,80,81,82]. Additionally, this association has been highlighted in other relevant publications [15,48]. However, while there is growing evidence that common genetic pathways may underlie both cancer and male infertility, the precise molecular mechanisms connecting these conditions remain to be fully elucidated and require further research.

GO biological process analysis also identified enriched terms related to cell proliferation, a key process in cancer development [81]. Additionally, the terms tube development and tissue development were enriched, highlighting their vital roles in the formation and function of the male reproductive system. Tube development involves the formation of crucial tubular structures, such as the seminiferous tubules, efferent ducts, and vas deferens, which are essential for sperm production, maturation, and transport [83]. Moreover, microtubules, as the primary components of the cytoskeleton, are vital for organelle transport and cell division during spermatogenesis, ensuring the functional capacity of sperm. Microtubule-based structures, such as the axoneme and manchette, are crucial for the proper formation of the sperm head and tail as well [84]. In parallel, tissue development encompasses the differentiation and maturation of testicular tissues, which are critical for spermatogenesis [85]. Disruptions in these developmental pathways can lead to structural abnormalities, impairing sperm production and contributing to various forms of male infertility, including asthenozoospermia.

Finally, the gene ontology (GO) analysis for cellular components highlighted the significant association of the Wnt signalosome, transcription factor AP1, and the β-catenin destruction complex with male infertility. The Wnt signaling pathway is a fundamental and highly conserved cascade involved in regulating various cellular processes, such as cell proliferation, differentiation, migration, and apoptosis [86]. It also plays a crucial role in sperm maturation and germ cell development, and its dysregulation can result in defective spermatogenesis and germ cell loss, potentially mediated by noncoding RNAs, as observed in mouse models [87]. Furthermore, the β-catenin destruction complex is a key regulator of Wnt signaling intensity, and its dysfunction has been linked to impaired spermatogenesis [87,88,89]. Additionally, the AP-1 family of transcription factors is important for regulating gene expression within Leydig cells, contributing to Leydig cell proliferation, steroidogenesis, and cell-to-cell communication [90]. Studies also indicate that this transcription factor is involved in the Sertoli-cell-mediated control of germ cell apoptosis [91]. Thus, the interplay between these factors and the Wnt signaling pathways is essential for maintaining normal reproductive functions, and disruptions in this network can contribute to conditions such as asthenozoospermia. Furthermore, the GO analysis identified intermediate-density lipoprotein (IDL) as the most enriched term for cellular components, pointing to an emerging area of research exploring the link between lipid metabolism and male infertility. Recent studies suggest that certain lipid traits, including low-density lipoprotein (LDL) and triglycerides, may be associated with an increased risk of male infertility [92]. However, the specific effects of IDL on male reproductive health remain to be fully elucidated and warrant further investigation.

### 3.3. Common Variants and lncRNAs

In this study, several prioritized variants with functional significance were found to affect both the structure of lncRNAs and their interactions with miRNAs, suggesting a potentially important role in male infertility. These variants, listed in Table 2, warrant further investigation to determine their effects on lncRNA function. Notably, one of the variants is mapped to *lnc-AGPAT5-1*, an lncRNA with an unexplored role and function. This same variant disrupts a substantial number of miRNA interactions, leading to the loss of 90 miRNA binding sites, which may have significant regulatory implications.

Additionally, among the variants disrupting lncRNA–miRNA interactions, multiple prioritized variants were identified for specific lncRNAs, as shown in Table 3. This suggests that these lncRNAs may be more prone to deregulation, underscoring their potential significance in the etiology of male infertility. However, further research is needed to validate these findings.

### 3.4. Male Infertility and Cancer—A Potential Association

In this study, KEGG pathway analysis revealed a significant association between the gene targets of affected miRNAs and cancer, while GO analysis identified several pathways shared between male infertility and cancer. Previous studies have indicated that infertile men are at a higher risk of developing cancer, suggesting the presence of shared molecular pathways [79,80,81,82]. Additionally, this association has been highlighted in other relevant publications [15,48].

To further investigate this connection, we performed an integrative analysis of KEGG pathways and reviewed the literature. Table 4 presents pathways shared between male infertility and cancer based on the findings of this study and the identified genes. Interestingly, many of these pathways exhibit opposite patterns of regulation: pathways upregulated in cancer tend to be downregulated in male infertility and vice versa. This opposing regulation reflects the distinct yet interconnected biological demands of these two conditions. In cancer, upregulation of pathways such as PI3K-Akt, MAPK, and cell cycle signaling drives unchecked cellular proliferation, evasion of apoptosis, and sustained survival under stress, hallmarks of oncogenesis [93,94,95]. Conversely, in male infertility, the downregulation of these same pathways often manifests as impaired cell cycle progression, increased germ cell apoptosis, and reduced survival of spermatogonial stem cells, contributing to disrupted spermatogenesis [96,97,98,99].

This inverse regulation underscores the divergent cellular priorities of these conditions. Cancer cells hijack these pathways to sustain their growth and evade cell death, while in male infertility, the same pathways, when dysregulated, fail to support the tightly regulated environment required for healthy spermatogenesis. These findings highlight the context-dependent nature of molecular pathways and emphasize the need for further research to unravel the functional consequences of their regulation in these distinct pathologies.

### 3.5. Noncoding Regions as Biomarkers

The use of noncoding RNAs (ncRNAs) as biomarkers for the detection and prognosis of various diseases has emerged as one of the most promising advances in molecular diagnostics [110,111,112,113]. NcRNAs are robust and noninvasive biomarkers that can be detected in biofluids such as blood, urine, and semen. Their differential expression patterns in pathological conditions make them ideal candidates for diagnostic purposes. Long noncoding RNAs (lncRNAs), in particular, demonstrate a tissue-specific expression profile, further enhancing their utility as biomarkers [114].

The application of ncRNAs as biomarkers is extensively studied in oncology. For instance, AFAP1-AS1 is an lncRNA that has been identified as a novel molecular marker for predicting tumor progression and metastasis in various cancers, including esophageal and colorectal cancers [115]. One of the most prominent examples of lncRNAs in clinical practice is also PCA3 (Prostate Cancer Antigen 3), an FDA-approved biomarker for prostate cancer diagnosis, which outperforms traditional PSA testing in specificity [116].

Beyond oncology, ncRNAs also hold potential in other disease contexts. Variants in noncoding regions are increasingly recognized for their predictive value, as studies reveal that a substantial portion of disease heritability lies within noncoding regions of the genome, which constitute the majority of the human genome [117]. For example, noncoding variants are implicated in neurodegenerative disorders such as Alzheimer’s disease and Parkinson’s disease, where they regulate genes critical for neuronal function and disease progression [118].

In the context of male infertility, numerous coding regions have been characterized for their essential roles in spermatogenesis, with mutations in these genes often leading to infertility. Specifically in asthenozoospermia, Lu et al. (2020) [22] identified a number of differentially expressed mRNAs in affected patients, underscoring the significance of coding regions in this condition. Further highlighting their importance, a recent study revealed variants in oxidative phosphorylation genes in asthenozoospermic men, demonstrating their potential as biomarkers [23]. In this context, as noncoding regions gain increasing attention for their regulatory roles, the findings of this study on noncoding regions can be considered complementary in a broader effort to fully understand asthenozoospermia. By integrating whole-genome sequencing (WGS) and RNA-seq data, we aimed to identify noncoding regions with biomarker potential, offering new perspectives on the condition that can be studied in combination with previous findings. Therefore, it is crucial to holistically study both coding and noncoding regions to uncover the underlying mechanisms and potentially identify novel biomarkers that, when combined with clinical data, could enhance the prediction and management of diseases and pathological conditions such as asthenozoospermia.

### 3.6. Limitations

Despite the valuable insights gained, this study has several limitations that must be acknowledged. We acknowledge that the limited sample size in our study may constrain the statistical power of our analyses and the generalizability of our findings. This is an important factor to consider when interpreting our results, as conclusions drawn from a small cohort may not fully reflect the broader population. To address this, we strongly encourage future research to involve larger and more diverse cohorts. Such studies would not only validate and replicate our observations but enhance the overall reliability and applicability of the findings. Genome-wide association studies (GWAS) could be particularly useful in validating the association of the reported variants with male infertility. However, despite this limitation, our study offers valuable preliminary insights into the distinct genomic profiles of asthenozoospermia and normozoospermia. This contribution is particularly significant given the scarcity of studies in this field employing next-generation sequencing technologies to examine these specific conditions. Additionally, it is worth noting that much of the existing research on male infertility and WGS also relies on relatively small cohorts [48,119,120]. This reflects a broader challenge within the field, emphasizing the need for larger-scale studies to advance our understanding on male infertility. It is also important to highlight that our study was based solely on recruited volunteers, which posed significant logistical challenges. Recruiting participants with only a single semen abnormality, such as asthenozoospermia, is particularly difficult, further contributing to the limited sample size.

Another limitation of our study is the use of pooled DNA samples for whole-genome sequencing, with two pools representing normozoospermic individuals and one pool representing asthenozoospermic individuals. Pooling was chosen because of logistical and financial constraints, as well as to enable the inclusion of a greater number of participants within the scope of the study. By combining DNA from multiple individuals into pools, we aimed to balance the representation of individuals in each group and maximize the breadth of our analysis. While pooling is a widely used approach in exploratory studies to optimize resources, it may compromise biological and technical variability compared with sequencing individual samples. This limitation reduces the ability to capture interindividual differences and may affect the generalizability of the findings. Despite these challenges, our study provides valuable preliminary insights into the genomic differences between normozoospermic and asthenozoospermic groups. These findings serve as an important foundation for future research, including larger-scale investigations with individual sample sequencing, which can validate and expand upon our observations.

One limitation of this study is the use of the hg19 genome assembly, which, while widely used and compatible with many bioinformatic tools, provides less comprehensive coverage of noncoding regions than more recent assemblies such as the GRCh38 (hg38) or the telomere-to-telomere (T2T) assembly. This may result in the exclusion of certain noncoding RNA genes that are annotated in these newer versions. More specifically, based on publicly available comparisons, it is estimated that approximately 15–20% more noncoding RNA genes are annotated in hg38, and even more in the T2T assembly. Thus, future studies based on newer versions could enhance the detection of additional noncoding elements and improve the overall depth of the analysis.

Additionally, our analysis was based on bioinformatics predictions, underscoring the need for functional experiments to confirm the effects of these variants on the structure and function of lncRNAs, as well as their downstream impact on miRNA and mRNA interactions. We made efforts to address these limitations by utilizing multiple databases and applying a stringent filtering process to enhance the robustness of our variant analysis. Nonetheless, further research involving in vitro and in vivo functional assays is critical to validate our findings and clarify the underlying mechanisms. Such validations could not only support the use of these variants as potential biomarkers but pave the way for developing targeted therapeutic strategies for managing male infertility, especially in cases of asthenozoospermia.

Finally, we did not include variants in coding regions. Our primary focus on long noncoding RNAs (lncRNAs) stems from their emerging role as critical regulators in various biological processes, including spermatogenesis and fertility [33]. Unlike protein-coding genes, lncRNAs remain underexplored in the context of male infertility, despite increasing evidence of their influence on gene expression, chromatin organization, and cellular signaling pathways [15]. By prioritizing lncRNAs, we aimed to identify not only differentially expressed lncRNAs but unique variants in lncRNA regions that are specific to asthenozoospermic patients. These unique variants represent an additional layer of potential biomarkers, as they are found exclusively in infertile individuals and may reflect disease-specific molecular alterations. Furthermore, biomarkers based on lncRNAs hold significant promise due to their tissue-specific expression, stability in biofluids, and ability to reflect specific pathophysiological states [121]. While we recognize the importance of protein-coding variants in male infertility, we intended to complement existing knowledge by highlighting the underexplored contributions of lncRNAs and prioritizing candidates for further functional validation.

### 3.7. Future Directions

Our study provides valuable insights into the genomic and transcriptomic alterations associated with asthenozoospermia; however, there are several important areas for further investigation. At first, there remains a need for further experimental validation to establish the functional significance of the identified genetic variants and differentially expressed long noncoding RNAs (DE lncRNAs). A particularly promising approach would involve analyzing the simultaneous occurrence of genetic variants and DE lncRNAs in the same samples to better understand their interplay and potential contribution to male infertility. These efforts will not only validate our current findings but provide a more comprehensive understanding of the molecular mechanisms underlying asthenozoospermia. We believe that these additional steps will enhance the translational potential of our findings, paving the way for the development of novel biomarkers for male infertility.

An important direction for future research is also the analysis of DE lncRNAs identified through our prioritization analysis in spermatozoa of asthenozoospermic men. Seminal plasma exosomes are well-established as key carriers of ncRNAs to spermatozoa [122]. Beyond this, seminal exosomes are critical for cell-to-cell communication, maintaining sperm motility and survival in the female reproductive tract, supporting spermatogenesis, and affecting male reproductive health [123]. Investigating the expression levels of prioritized DE lncRNAs directly in spermatozoa, except for seminal plasma exosomes, as performed in this study, could provide critical evidence to establish a direct link between exosomal lncRNAs and their functional contributions to male infertility. This approach would not only confirm the biological relevance of these lncRNAs in the context of asthenozoospermia but potentially help uncover molecular mechanisms that connect exosomal cargo to the regulation of sperm health.

Furthermore, as our study included mainly computational tools, a more comprehensive understanding of lncRNA–miRNA–mRNA regulatory networks in male infertility requires further experimental validation. LncRNA–miRNA–mRNA interactions play a critical role in regulating gene expression [45] and can significantly impact fertility by influencing key processes such as spermatogenesis, sperm motility, and overall sperm function [8]. One promising direction for future research is the analysis of prioritized lncRNAs, miRNAs, and their target mRNAs in both seminal exosomes and spermatozoa of asthenozoospermic men. This analysis would provide critical evidence to confirm the disruption of lncRNA–miRNA interactions and their downstream effects on gene expression. Elucidating the mechanisms underlying lncRNA–miRNA–mRNA regulation could reveal how genetic variants in lncRNAs identified through whole-genome sequencing impact molecular pathways associated with sperm motility and fertility.

## 4. Materials and Methods

### 4.1. Whole-Genome Sequencing (WGS): Biological Samples and Analysis—Identification of Exclusive Variants

We collected blood and semen samples from Greek volunteers who agreed to participate in the Spermogene (Fertilaid) research program (Grant Τ1ΕΔK-02787), conducted in collaboration with the Embryolab IVF Unit in Thessaloniki, Greece. The study was approved by the Ethics Committee of the University of Thessaly, and all participants provided written consent after being fully informed of the program and its objectives.

All volunteers underwent a comprehensive andrological examination and completed a questionnaire detailing their medical and reproductive history. Based on this information, we excluded patients with known causes of male infertility, such as reproductive tract infections, systemic diseases, varicocele, cryptorchidism, etc., and men with genetic findings such as Y microdeletions, chromosomal abnormalities, etc. After these initial evaluations, semen samples from each participant were analyzed according to the fifth edition of the World Health Organization (WHO) manual for the examination and processing of human semen (available at: https://apps.who.int/iris/handle/10665/44261, accessed on 17 November 2024). Semen samples were obtained by masturbation after a period of abstinence of two to three days, then left to liquefy at 37 °C for 30 min before examination. All semen analyses were performed by a certified andrology laboratory, where several key parameters were evaluated, including sperm volume, concentration, motility, morphology, etc. WHO guidelines were employed for the processing and classification of human sperm. Specifically, the samples were classified using the seminogram results and reference values stipulated in the WHO guidelines. Based on these criteria, the semen samples were categorized into normozoospermic and asthenozoospermic groups. It should be noted that all samples in the normozoospermic group were derived from men with proven fertility (previous pregnancy), as indicated by the questionnaire they completed.

Then, we extracted DNA from the blood samples of the subjects using the PureLink Genomic DNA Mini Kit (Invitrogen, Waltham, MA, USA) as per the manufacturer’s guidelines. We measured the DNA concentration spectrophotometrically with the Qubit 2.0 fluorometer and the Qubit dsDNA BR Assay (Invitrogen, Waltham, MA, USA), while DNA quality was evaluated using agarose gel electrophoresis. Only high-quality samples were used for subsequent analysis. Purified DNA was stored at −20 °C before use. Subsequently, we prepared three sequencing pools. DNA from ten individuals characterized as normozoospermic was divided into two pools, each containing DNA from five individuals. Additionally, another pool was created using genetic material from five asthenozoospermic individuals. The DNA samples in each pool were combined in equal amounts, resulting in a concentration of 100 ng/uL and a total amount of 2 mg.

After preparing the samples, whole-genome sequencing was performed by Novogene, Cambridge (UK). Genomic DNA, prepared according to the method described above, was used to construct whole-genome sequencing libraries. Following normalization and strict quality control procedures, the libraries were sequenced using an Illumina HiSeq 3000 platform (Illumina Inc., California, USA) with 100 bp paired-end reads. The sequencing coverage reached an average of 30×. The sequencing data produced were subjected to standard bioinformatics analysis. For this procedure, we first assessed the quality of the reads using FASTQC (http://www.bioinformatics.babraham.ac.uk/projects/fastqc/, accessed on 17 November 2024) and then removed low-quality reads (with a minimum PHRED score of 30) and adapter sequences using Trimmomatic (v0.39) [124]. The remaining reads were aligned to the human reference genome (GRCh37/hg19), retrieved from the Ensembl database [125], using the Burrows–Wheeler aligner (BWA, version 0.7.17) [126]. To remove duplicated reads, we employed Picard tools, followed by converting SAM files to BAM files using SAMtools (v1.19.2) [127]. At this stage, we merged individual BAM files for normozoospermic pools into a single file to analyze all the variants found in normozoospermic men. Subsequently, we used freeBayes (v1.3.7) [128] for variant calling, and the results were saved in variant call format (VCF). Then, we employed BCFtools version 1.17 [127] to compare VCF files from normozoospermic and asthenozoospermic individuals in order to identify unique variants exclusive to each group, found only in normozoospermic or only in asthenozoospermic. Finally, we annotated the unique variants exclusive to either normozoospermic or asthenozoospermic individuals using the VEP software (https://www.ensembl.org/Tools/VEP, accessed on 17 November 2024) to retrieve biological information and assess the variants’ effects and disease-causing potential.

### 4.2. RNA Sequencing—Identification of Differentially Expressed (DE) lncRNAs Between Asthenozoospermic and Normozoospermic Men

For RNA analysis, data were obtained from the publication by Lu et al. (2020) [22] to identify differentially expressed lncRNAs between normozoospermic and asthenozoospermic individuals. In summary, total RNA was extracted from the seminal plasma exosomes of 25 men diagnosed with asthenozoospermia and 25 healthy men with normal sperm parameters. The quality and quantity of RNA were evaluated, and ribosomal RNA was depleted to allow for accurate analysis of the lncRNAs. After library preparation, sequencing was performed using the Illumina HiSeq platform, generating high-throughput data. Data processing and quality control were conducted to ensure data integrity. All RNA-seq analyses were performed using R version 3.6.1. Differential expression was determined using the DESeq2 package (version 1.24.0) to identify differences in gene expression levels between the asthenozoospermic and the normozoospermic group. Additional statistical and visualization analyses were performed using ggplot2 version 3.2.1. Genes with a *p*-value ≤ 0.05 and an absolute log_2_ fold change (FC) ≥ 1 were considered differentially expressed.

### 4.3. Prioritization of Variants Based on Their Impact on lncRNA Functionality, Structure, and miRNA–lncRNA Interactions

After identifying unique variants in asthenozoospermic men using whole-genome sequencing and DE lncRNAs between asthenozoospermic and normozoospermic men using RNA sequencing, the two datasets were combined. Only the unique variants identified in asthenozoospermic men and located in DE lncRNAs were selected for further analysis, since variants in lncRNAs can affect their structure, functionality, and role by disrupting their interactions with miRNAs, which in turn can impact gene regulation. Through these mechanisms, variants in lncRNAs can contribute to complex conditions, such as male infertility, while also serving as important biomarkers that combine unique expression profiles and variants observed only in infertile men. Therefore, subsequently, we applied a series of filters to prioritize the identified variants.

#### 4.3.1. Prioritizing Variants Based on Functionality

At first, the unique variants mapped in DE lncRNAs were assessed for their potential functional roles using two databases. The 3DSNP v2.0 database [26] was utilized to estimate the potential functional impact of each variant, selecting those with a 3DSNP score greater than 20, which indicates a probable functional role. 3DSNP v2.0 provides annotation for human noncoding variants, including information about 3D-interacting genes, enhancer and promoter states, transcription factor binding sites, etc. Additionally, RegulomeDB 2.2 [25] was employed to rank these variants based on functional significance, prioritizing those with a rank between 1a and 2c. RegulomeDB integrates data on regulatory elements such as transcription factor binding and DNase hypersensitivity sites, enabling effective filtering of variants with potential regulatory impacts on gene expression and functionality [25]. Variants with potential functionality were prioritized based on a 3DSNP score greater than 20 and a RegulomeDB rank between 1a and 2c.

#### 4.3.2. Prioritizing Variants Based on Structural Impact

For structural assessment of unique variants mapped to DE lncRNAs, the lncRNASNP v3 database [27] was used to analyze how each variant may alter the secondary structure of lncRNAs. LncRNASNP v3 is a comprehensive database for lncRNAs that provides computational predictions on how specific single-nucleotide polymorphisms (SNPs) can affect RNA structure, helping to pinpoint structural alterations that could impair lncRNA functionality [27]. Only variants with a *p*-value < 0.2 were prioritized, as they are more likely to impact structural stability or folding. It should be noted that this threshold aligns with the recommendations provided by the database creators.

#### 4.3.3. Prioritizing Variants Based on miRNA–lncRNA Interaction Disruption and Investigation of Mechanisms and Pathways Affected

To determine whether any variants might disrupt miRNA–lncRNA interactions, we utilized lncRNASNP v3 [27]. This database also includes information about SNPs that are likely to affect miRNA binding sites in lncRNAs, providing information on how genetic changes could disrupt or modify the lncRNAs’ regulatory roles by altering miRNA-binding affinity. More specifically, it provides details about the loss or gain of interaction sites, as well as the miRNAs that are affected. Exclusive variants on DE lncRNAs with evidence of impacting these interactions were prioritized for further analysis. Once variants potentially disrupting miRNA–lncRNA interactions were identified, the next step was to determine the downstream targets of these miRNAs using miRTargetLink 2.0 [28]. In this step, we selected only experimentally validated targets. Subsequently, we performed gene ontology (GO) [29,30] and KEGG pathway [31] analyses using ShinyGO version 0.81 [32] to elucidate the biological pathways and processes potentially impacted by these interactions, shedding light on how such regulatory changes may contribute to the phenotype of asthenozoospermia.

## 5. Conclusions

In this study, we identified and prioritized several unique variants mapped to DE lncRNAs, found exclusively in asthenozoospermic men. These variants are predicted to alter lncRNA structure and disrupt lncRNA–miRNA–mRNA interactions, potentially affecting key regulatory pathways involved in male infertility. With the above mechanisms, the identified variants may influence crucial biological processes, such as spermatogenesis and sperm motility, underscoring their potential significance in the etiology of male infertility. Furthermore, the enriched pathways identified in our analysis, including Wnt signaling, phosphatase binding, and cell proliferation, have been previously implicated in male reproductive disorders, reinforcing the potential impact of these prioritized variants. Notably, the exclusivity of variants to asthenozoospermic men suggests also a promising role as potential biomarkers for diagnosing or predicting this specific condition.

It should be noted also that this study represents a complementary approach to previously published research examining the role of mRNAs in asthenozoospermia. Variants in coding regions of the genome, as well as their differential expression, are well-characterized and known to provide valuable insights into this condition. In the present study, however, we focused on noncoding regions to holistically address the issue and explore their role, which has been gaining increasing attention in recent years. By integrating findings from both coding and noncoding regions, we aim to provide a more comprehensive understanding of asthenozoospermia, facilitating a holistic interpretation of its underlying mechanisms.

Overall, given the limited number of studies utilizing whole-genome sequencing (WGS) data to explore the role of variants in noncoding RNAs (ncRNAs) in male infertility, our findings provide valuable insights. By pinpointing specific SNPs and emphasizing lncRNAs that may contribute to the pathogenesis of male infertility, this study offers a roadmap for future research, highlighting lncRNAs and variants that warrant further investigation. Ultimately, such studies can advance our understanding of the genetics underlying complex diseases and traits like asthenozoospermia and pave the way for new diagnostic and therapeutic strategies.

## Figures and Tables

**Table 1:** Prioritized variants with an impact on lncRNA structure according to lncRNASNP v3.

Variants	Gene	Gene ID (Ensembl)	Transcripts	*p*-Value
rs35710229	*NKX2-1-AS1*	ENSG00000253563	NONHSAT036440.2	0.0851
rs67786346	*RUVBL1-AS1*	ENSG00000239608	NONHSAT091752.2	0.1978
			NONHSAT194440.1	0.0704
rs113300435	*NEXN-AS1*	ENSG00000235927	NONHSAT226787.1	0.1152
			NONHSAT004050.2	0.1108
rs2276699	*LINC01914*	ENSG00000234362	NONHSAT070300.2	0.1230
rs2951831	*lnc-AGPAT5-1*	ENSG00000245857	NONHSAT124792.2	0.1733
			NONHSAT254767.1	0.1580
			NONHSAT254769.1	0.1568
			NONHSAT124793.2	0.1584
			NONHSAT254768.1	0.1499
			NONHSAT215358.1	0.1446

**Table 2:** Prioritized variants that affect both lncRNA structure and lncRNA–miRNA interactions.

Variant	lncRNA	*p*-Value (Effect on Structure)	miRNA Gain	miRNA Loss
rs35710229	*NKX2-1-AS1*	0.0851	0	6
rs2951831	*lnc-AGPAT5-1*	0.1733 0.1580 0.1568 0.1584 0.1499 0.1446	6	90

**Table 3:** LncRNAs with multiple prioritized variants disrupting lncRNA–miRNA interactions.

**lncRNAs**	**Variants**	**miRNA Gain**	**miRNA Loss**
*PSMA3-AS1*	rs10145437	5	0
	rs7153897	0	14
*LINC00667*	rs1201525	0	3
	rs35455334	3	0
*AATBC*	rs66472331	3	3
	rs73367288	2	2

**Table 4:** Shared pathways between male infertility and their regulation according to KEGG pathways and search of the literature.

Pathway	Genes	Male Infertility	Cancer
Wnt signaling pathway	*SOX4*, *MYC*	Downregulated [100]	Upregulated [101]
TGF-beta signaling pathway	*SOX4*, *STAT3*	Dysregulated (both) [102]	Dysregulated (both) [103]
Apoptosis	*TP53*, *BCL2*, *MAPK3*, *MYC*	Upregulated [104]	Downregulated [105]
Cell cycle	*TP53*, *CDK4*, *MAPK3*, *MYC*	Dysregulated (both) [96,97]	Upregulated [95]
PI3K-Akt signaling pathway	*CDK4*, *BCL2*, *AKT1*, *MYC*	Downregulated [99]	Upregulated [93]
FoxO signaling pathway	*BCL2*, *AKT1*	Upregulated [106]	Downregulated [107]
MAPK signaling pathway	*MAPK3*	Downregulated [98]	Upregulated [94]
p53 signaling pathway	*TP53*	Upregulated [108]	Downregulated [109]

## Data Availability

Whole-genome sequencing data of normozoospermic men used in this study are available through SRA (BioProject ID PRJNA875412, http://www.ncbi.nlm.nih.gov/bioproject/875412, accessed on 17 November 2024).

## Supplementary Materials

The following supporting information can be downloaded at: https://www.mdpi.com/article/10.3390/ncrna11010004/s1, Table S1: List of DE lncRNAs; Table S2: List of unique variants mapped onto DE lncRNAs; Table S3: RegulomeDB ranks and 3DSNP scores of identified variants; Table S4: SNPs that cause miRNA target gain/loss according to lncRNASNP v3; Table S5: Common genes targeted by the affected miRNAs according to miRTargetLink 2.0.
